# Gaps in access to pulmonary hypertension care and opportunities for improvement: a multi-site qualitative study

**DOI:** 10.1186/s12890-025-03817-4

**Published:** 2025-07-28

**Authors:** Kari R. Gillmeyer, Sara Shusterman, Seppo T. Rinne, A. Rani Elwy, Renda Soylemez Wiener

**Affiliations:** 1 Center for Health Optimization & Implementation Research, VA Boston Healthcare System, Boston, MA USA; 2 The Pulmonary Center, Boston University Chobanian & Avedisian School of Medicine, Boston, MA USA; 3 Geisel School of Medicine at Dartmouth, Lebanon, NH USA; 4 Department of Psychiatry and Human Behavior, Warren Alpert Medical School of Brown University, Providence, RI USA; 5 Center for Health Optimization & Implementation Research, VA Bedford Healthcare System, Bedford, MA USA

**Keywords:** Delayed diagnosis, Health services accessibility, Patient-centered care, Pulmonary hypertension

## Abstract

**Background:**

Pulmonary hypertension (PH) is a progressive disease leading to right heart failure and early mortality. Early recognition of the disease and timely initiation of PH-specific therapies for qualifying PH subgroups are crucial for improving patient outcomes. Yet delays in diagnosis and treatment of PH persist. We aimed to explore patient and provider perspectives on access to and timeliness of PH care across the patient’s entire health journey from symptom onset through follow-up care.

**Methods:**

We conducted a multi-site qualitative study at three expert PH centers in the United States. We interviewed 41 key informants including 21 patients and 20 providers (physicians, physician assistants, PH pharmacists, and PH nurses). Guided by a conceptual model of the care continuum adapted to PH, we analyzed transcripts using a directed content analysis with both deductive (based on our conceptual model) and inductive coding.

**Results:**

We found barriers to timely access to PH care along the entire care continuum from symptom onset through receiving longitudinal PH care. Geographic barriers to care, limited non-expert PH knowledge, dismissal of patient’s symptoms by providers, limited PH expert availability, and inadequate insurance coverage of PH medications emerged as the most prominent barriers to PH care access. Participants offered clear and specific solutions to address these care gaps, including establishing telementoring models to improve non-expert PH knowledge, building relationships between PH experts and community providers to bolster referral networks, leveraging technology to mitigate geographic barriers, and building satellite sites to expand access to PH experts.

**Conclusions:**

Patients with PH experience significant barriers to receiving timely PH care along their entire health journey. Comprehensive transformations to PH care delivery and health policies are needed to mitigate delays and improve quality of care for patients living with this disease.

**Supplementary Information:**

The online version contains supplementary material available at 10.1186/s12890-025-03817-4.

## Background

Pulmonary hypertension (PH) is a progressive disease characterized by elevated pressures within the pulmonary vasculature that can lead to right heart failure and early mortality [1]. Key to improving outcomes for patients with PH is early recognition of the disease and prompt initiation of PH-specific therapies for patients with qualifying PH subgroups such as pulmonary arterial hypertension (PAH), chronic thromboembolic PH (CTEPH), and PH associated with interstitial lung disease [1–3]. Guidelines recommend early referral to expert PH centers for patients with suspected or confirmed PAH or CTEPH and any patient who may be a candidate for PH therapies, as outcomes for patients cared for within these centers are improved [4–7]. Yet, despite these recommendations and efforts to raise awareness of the disease [8], the time from symptom onset to diagnosis among patients with PAH and CTEPH has not improved over the past 30 years [9–11] and delayed referrals to expert PH centers are common [12, 13]. Furthermore, patients may experience additional delays in being initiated on PH treatment even after they have received a PH diagnosis [14].

Prior quantitative studies have identified factors associated with diagnostic delays in PH, including older age and presence of comorbid respiratory and cardiac diseases [15–17]. Additionally, prior qualitative work has explored patient perspectives on drivers of diagnostic delays [18–20]. Yet, existing literature has not simultaneously explored patient and provider perspectives on access to or timeliness of PH care through a patients care journey, information that could pinpoint problems across the care continuum and offer more comprehensive solutions to improving PH care.

Drawing from their deep and diverse experience in PH care delivery, expert PH centers offer a rich environment in which to explore the context of diagnostic delays and barriers to PH care access. We therefore sought to integrate provider and patient perspectives on timely access to PH care across the PH care continuum, within and across expert PH centers in the United States.

## Methods

### Overview

**Fig. 1:**
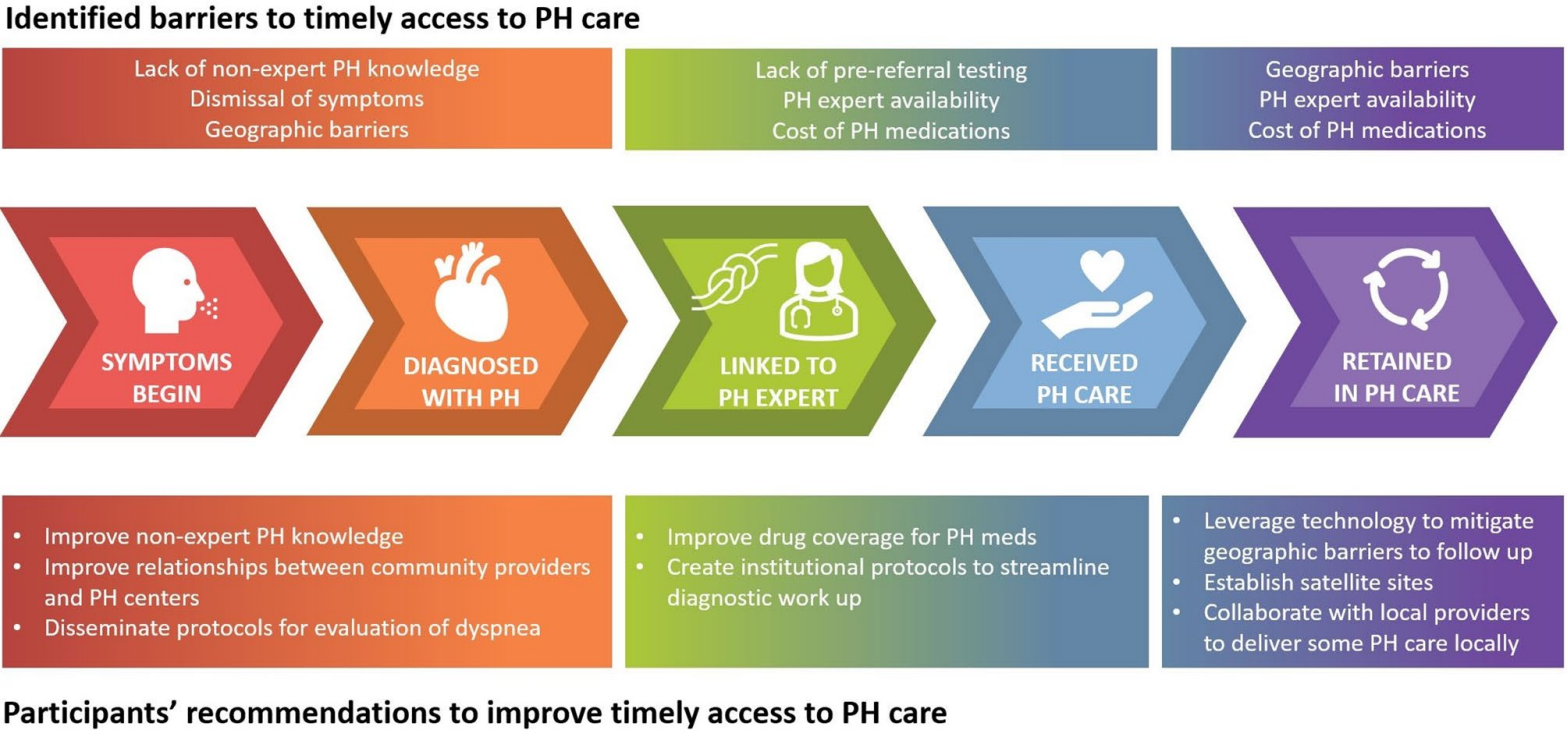
Barriers to timely access to pulmonary hypertension care and proposed solutions along the pulmonary hypertension care continuum. Note that for some patients, Step 3 (Linked to PH Expert) may occur before Step 2 (Diagnosed with PH). PH = pulmonary hypertension

We conducted a multi-site qualitative study exploring provider and patient perspectives on access and timeliness of PH care at three expert PH centers in the United States. PH centers were chosen based on diversity of patient populations served: one site serves a higher economic patient population while the other two predominantly care for underserved populations. We adapted a conceptual model of the care continuum in HIV [21] to PH (Fig. 1) in order to examine PH care access across the entire trajectory of a patient’s health journey, from symptoms onset through longitudinal care.

### Participant recruitment

We used purposeful sampling [22] to recruit provider participants at each site. We identified section chiefs or other relevant leadership at each site through institutional websites and asked them to identify PH care providers at their sites, such as pulmonologists, cardiologists, advanced practice providers (physician assistants or nurse practitioners), pharmacists, and/or nurses. We asked provider participants to identify additional relevant informants for interviews [23]. We focused our recruitment efforts on providers most integrally involved in PH care at each site regardless of discipline. We recruited a convenience sample of patients at Sites A and C by (1) asking PH providers to recommend patients for interview, and (2) posting flyers within PH clinics instructing interested patients to contact the study team. We were unable to recruit patients at Site B due to Institutional Review Board (IRB) restrictions. Inclusion criteria for patient participants were age ≥ 18, English-speaking, diagnosis of any subgroup of PH, and receipt of PH care at the included site. Per guidance on sample sizes for qualitative research [24], we targeted at least 17 participants per group. Table 1 shows participant characteristics. Most (52%) patient participants were female and most (90%) were on PH treatment at the time of the interview. The most common PH subgroups were Group 1 PH (38%) followed by Group 5 (24%).

**Table 1:** Characteristics of study participants by site

	Site A	Site B	Site C
Providers interviewed^*^			
Pulmonologists	7	5	3
Cardiologists	1		
Pharmacists		1	
PH nurses		1	1
Advanced practice providers	1		
Patients interviewed^†^	10		11
Female	5		6
Pulmonary Hypertension WHO Group			
Group 1	4		4
Group 2	1		1
Group 3	2		1
Group 4	1		2
Group 5	2		3
Total participants	19	7	15

All data presented as n

*WHO* World Health Organization

^*^Site A did not have pharmacists or nurses directly involved in PH care, Site B did not have cardiologists or advanced practice providers involved, and Site C did not have cardiologists, pharmacists, or advanced practice providers involved in PH care

^†^We were unable to recruit patients at Site B due to IRB restrictions

### Data collection and analysis

Between August 2022 and November 2023, a pulmonologist with experience and training in qualitative research (KRG) conducted semi-structured telephone interviews with 20 providers and 21 patients across the three sites. Audio-recordings were transcribed verbatim for analysis. Interviews explored each segment of the PH care continuum, from onset of symptoms to follow-up care (see interview guides in the Supplementary Material).

We conducted a directed content analysis [25] including both deductive (using an a priori coding framework developed from the HIV Care Continuum model) and inductive (open to unanticipated but important themes that emerge from the data) approaches. To ensure consistency, 20% of transcripts were double coded by two team members (KRG, SS) with frequent team meetings to discuss codes and ensure consensus. We systematically examined our data across all participants and sites to understand similarities and differences that occurred. We used NVivo 14 software to organize qualitative data.

## Results

We present our findings aligned with three segments of the care continuum: (1) reaching PH care after onset of symptoms, (2) initial evaluation and treatment initiation within the PH center, and (3) longitudinal care after diagnosis and treatment initiation. Subdomains within each segment are also identified and described. We present patient and provider perspectives together within each of these segments. Finally, we highlight participant recommendations to improve access and timeliness of PH care.

### Reaching PH care

Patients and providers alike noted significant delays in diagnosis and referral to expert care: *“There are tremendous delays of care…by the time [patients] make their way to [our center]*,* there’s been such a delay in care that they often have progressed disease” –* Physician, Site C.

Patients described a variety of ways in which they ultimately found a PH expert. While some patients were formally referred by their primary care providers or other specialists, others reached PH care through self-referral, personal connections to an expert, or recommendations from other patients: *“[My local pulmonologist] said*,* ‘I don’t know what it is.’ And I said*,* ‘I’m not gonna fool around with this.’ So*,* I did some research online and that’s how I wound up at [Site A]…I pursued it myself”* – Patient, Site A. Patients described the path to finding a PH expert as convoluted, noting “*This was not easily diagnosed ‘cause it took three pulmonologists to do it” –* Patient, Site A.

Providers agreed that patients reached them through both formal and informal mechanisms. Referrals were often facilitated by professional relationships between the referring provider and PH expert: *“I have former fellows who work out in the community and have an interest in PH and they send me patients. So*,* with some people*,* we have a personal connection”* – Physician, Site B.

Participants identified several drivers of diagnostic and referral delays, including (1) lack of non-expert knowledge about PH, (2) dismissal of symptoms by providers, and (3) geographic barriers to reaching PH care.

#### Lack of PH knowledge

Participants felt that gaps in PH-specific knowledge among non-experts led to delays in referral: “*A lot of patients get referred late in the course because community docs are not well versed in PH”* – Physician, Site A. These knowledge gaps may lead to delays in obtaining necessary testing: *“The gap between where [PH] experts are and where the initial assessments are is so vast…The consultants end up having to launch an exhaustive evaluation…which hasn’t been done very well at first…If you’re lucky*,* you have a provider that recognizes that the [pulmonary artery] pressures are elevated and doesn’t know what they’re doing*,* and they’ll refer in” –* Physician, Site A.

#### Dismissal of symptoms

Patients felt that providers often did not take their symptoms seriously, which contributed to delays: “*I tried to work this through with my PCP who did a cursory review of symptoms and then told me*,* ‘You’re fine. Stop pursuing this’*” – Patient, Site A. Patients felt that comorbidities such as obesity or mental health diagnoses contributed to the dismissal of symptoms: “*I was having trouble breathing while exerting myself and the usual suspects of*,* ‘You’re obese*,*’ and ‘You’re deconditioned*,*’ came up” –* Patient, Site A. Self-advocacy and advocacy by friends and family was often key to getting diagnostic tests performed or referrals placed: *“You have to fight for your rights…you have to be demanding when you really don’t feel good”* – Patient, Site C.

#### Geographic barriers to reaching care

Both providers and patients believed that geography affected access to PH care: “*I thank God every day that I’m not in the middle of Kansas. I got the best doctors in the world in [large city]”* – Patient, Site A. *“We have a significant percentage of people from rural communities that are coming here because of inability to get adequate PH care where they live”* – Physician, Site C.

### Evaluation within the PH center

#### Triage of new patients

Participants noted delays in new referrals being seen: “*There’s lots of patients coming in and out*,* so just getting the patient in for an evaluation…can be challenging…patients have to wait sometimes weeks to months to be seen”* – Physician assistant, Site A. Sites triaged referrals based on clinical urgency: *“We try to accommodate case-by-case to make sure a patient who needs to be seen soon will be seen…within a week or so”* – Physician, Site B.

#### Evaluation

Providers noted that the lack of preliminary testing done prior to referral contributes to diagnostic delays: *“What would streamline [referrals] is just simple education…that everybody who you suspect for PH should have an echocardiogram*,* because that…will expedite everything immensely”* – Provider, Site C. At one site, limited resources within the center contributed to delays: “*There are challenges with getting cath lab space….many times it depends on both availability of the [procedure] room*,* as well as availability of nurses and techs”* – Physician, Site C. Other sites felt that resources were adequate for timely evaluations: *“We can accommodate most patients very quickly*,* and I think the fact that we do all of our own right heart caths makes it even more streamlined and more efficient”* – Physician, Site A.

#### Initiation of PH treatment

Patients and providers alike noted significant delays in starting PH therapies after the diagnosis was made, primarily related to cost of medications and inadequate insurance coverage: “*[PH] medicines are ridiculously expensive…when patients are on [Medicaid]…getting PH meds is practically out the window because [Medicaid] won’t pay for them” –* PH nurse, Site C. While financial support programs are available to help offset the costs of PH medications, navigating the application process for these programs was often arduous for patients: “*Getting financial assistance has been extremely challenging…It took 63 phone calls between my insurance and the financial assistance people to finally get the [PH] meds in hand”* – Patient, Site A. The challenges of getting patients started on PH medications also sometimes extended to other therapies like home oxygen: *“Some O2 companies will offer it temporarily*,* but then they charge the patient…and [the patient doesn’t] have the money to pay for it”* – PH nurse, Site C.

### Longitudinal follow-up

Participants noted several challenges with maintaining timely access to care after diagnosis and treatment initiation, including (1) geographic access, (2) provider availability, and (3) ongoing access to PH treatment.

#### Geographic access

Providers noted that their referral networks were often vast, extending across states, which created challenges with follow-up care: *“Patients are coming two to three hours from where they live. So*,* that’s the challenge sometimes to try to get patients to come in”* – Physician assistant, Site A. Patients confirmed these challenges: *“Getting in and out of that place*,* it’s a nightmare”* – Patient, Site A. Geographic barriers to care were even more pronounced among patients with lower economic means: *“It’s difficult for [my patients] to get into [large city] very often because they…tend to be a lower socioeconomic demographic and transportation is very challenging…a lot of these patients don’t end up getting care quite often”* – Physician, Site B.

#### Provider availability

Patients noted that availability of PH providers was limited: *“They’re booked to the gills”* – Patient, Site A. In the case of acute issues, however, patients felt that their needs were addressed: *“Sometimes my breathing becomes difficult and whenever I have issues with that…they squeeze me into [my PH provider’s] tight schedule”* – Patient, Site C. To overcome the lack of available appointments, some providers made themselves available to patients between clinic visits, often outside of business hours: *“I often give patients my cell phone number… I want them to let me know when things are going awry without hesitation”* – Physician, Site B. However, trying to compensate for lack of clinic availability took a toll on physicians: *“I’m getting to a breaking point… I’m often seeing like nine or ten patients with PH [which is] impossible in a four-hour session…My patients who are really active*,* I see every month…for things that a nurse practitioner could do*,* including medication teaching*,* diuretic management. …It’s one of the reasons that my clinic is so incredibly full”* – Physician, Site C. By contrast, other PH centers leveraged support from advanced practice providers to offload PH physicians’ schedules and address acute issues: *“Our [physician assistants] are being increasingly used to help us…see patients in between visits because a lot of us…are booked out for a minimum of three to four months”* – Physician, Site A.

#### Ongoing access to PH treatment

Even after PH treatment initiation, ongoing challenges with insurance coverage of medications and access to copay assistance persisted, sometimes disrupting PH therapy: *“I needed funding to pay the copay [for Ambrisentan]…About a year and a half later*,* they said…they could no longer fund the copay and I had to stop taking it”* – Patient, Site A. Providers agreed that the high cost of PH medications could hamper treatment adherence: “*It’s challenging to get a patient to be compliant with treatment if they can’t afford it”* – Physician assistant, Site A. Maintaining patients on therapy required ongoing intensive coordination among different parties: *“Access to drugs is…cumbersome because every patient has a unique insurance program. And you use combination therapies. …One drug comes from one specialty pharmacy. The other drug comes from a different specialty pharmacy…so navigating through the prior authorization process*,* it’s unbelievably time-consuming” –* Physician, Site B.

### Recommendations to improve PH care

Both patient and provider participants proposed recommendations to mitigate delays in PH care. Their recommendations, with representative quotes, are shown in Table 2 and mapped to the relevant step(s) in the care continuum in Fig. 1.

**Table 2:** Patient and provider recommendations to improve timeliness of and access to pulmonary hypertension care

Recommendation	Representative patient quotes	Representative provider quotes
Improve non-expert knowledge about PH, potentially through PH-specific telementoring programs	*“My biggest recommendation is to work with primary care physicians*,* internal medicine who see patients at the very beginning and to educate them…The primary care and the internal medicine doctors are really the key ‘cause you’re not gonna see a pulmonologist or a cardiologist until there’s something really wrong”* – Patient, Site A *“Everyone needs to be educated about the medications for pulmonary hypertension because I’ve gone to my [outside] pulmonologist and the nurse didn’t even know what the medications were…I think education on the disease and the medications for the disease is definitely in order”* – Patient, Site C	*“The fact of the matter is that there’s a huge cliff that everybody falls off between general practitioners and real experts in the field. The field has evolved so quickly and it’s so nuanced that it’s really challenging for people to follow”* – Physician, Site A “*One thing that might be helpful is to create something like an echo program for pulmonary hypertension*,* which is like an online hub-and-spoke model for education to try to reach out to pulmonologists and cardiologists that may be seeing those patients in the communities*,* to just increase awareness around PH and what is involved in diagnosing PH and treating it and the need for right heart cath and how to interpret said right heart cath. There’s a lot of pieces that seem to be missing out of the community”* – Physician, Site C
Improve relationships between community providers and PH centers through outreach programs to facilitate timely referrals		*“The physicians in our program [have done] outreach to community physicians and the avenue for referral patterns have been better over the years…Ever since we’ve been doing more outreach we’re not getting as many sicker patients…and we’re starting to see more earlier PH patients…so*,* I think it is working at least in some respect”* – Physician assistant, Site A *I think just better visibility would help…because I think a lot of people don’t know that we have a PH clinic still. I think just making it more visible so that people know who to send*,* where to send patients is an area that we can improve upon” –* Physician, Site B
Create and disseminate protocols for the evaluation of dyspnea	“*I guess maybe just a faster referral from the PCP to a pulmonologist for patients who have passed an EKG and have passed a pulmonary function test*,* but still complain of issues. I had trouble climbing stairs at one point and I knew this wasn’t right*,* but I didn’t get any referrals to a pulmonologist” –* Patient, Site A	*“We have a cardiopulmonary exercise program that’s pretty well established and there’s been a lot of work up from that group*,* sort of looking at the costs of diagnosis of unexplained dyspnea suggesting that referring patients for cardiopulmonary exercise testing earlier may expedite achieving a diagnosis” –* Physician, Site A
Expand insurance coverage of PH medications	*“There needs to be a change within with drug coverage*,* but that’s not something [Site A] can address*,* really.” –*Patient, Site A	
Create institutional protocols to streamline diagnostic work-up		*“Systems-wise*,* a lot of these things could be done on the same day. Echocardiogram*,* CT scan*,* PFTs… if there was a way to protocolize these tests being done at the same time*,* which is the crux of the evaluation for pulmonary hypertension*,* I think that it would infinitely expedite evaluation and start of treatment*,* if needed” –* Physician, Site C
Leverage technology (including virtual visits and electronic health records) to mitigate geographic barriers and maintain follow up	“*I’m retired now. But I was working*,* and I think it was very beneficial that I could make a televisit without having to lose a day of work travelling into [the city]. And I think it’s important too for people who have children at home or*,* someone they’re caring for*,* that this is more convenient” –* Patient, Site C	*“I think the big issue really is transportation. I have tried to incorporate televisits with a lot of our patients since COVID who live long distances away because I’d rather have them do a televisit than no visit at all”* – Physician, Site C *“I am trying to get it so that the [electronic health record] starts giving reports of patients who have cancelled or their appointments disappeared. That’s another way that we can try to keep up with those patients”* – Physician, Site C
Establish satellite sites to improve access	“*We live in [small town] and my pulmonologist is downtown at the hospital. And we have this gorgeous new facility [nearby]…[it would be] be nice if they could have a [PH] pulmonologist there” –* Patient, Site A	*“I think access and geography is big. I’ve especially seen it with one of my patients who would miss most of her appointments with me down at [Site B]. And now that I’m at [satellite site]*,* she misses only half as many as she used to [at main site] just because I think it’s much easier for her to get to”* – Physician, Site B
Develop a collaborative approach with local providers so some PH care can be delivered locally		*“We usually involve [the patient’s] outside pulmonary providers early*,* especially if [the patient is] from far away and it’s not easy for the patient to come in or the patient expresses hesitancy about trying to come into [city] for their follow up PH care*,* then we try to involve the pulmonologist or the cardiologist early in a collaborative treatment plan*,* creating a treatment plan together and then assessing their comfort in starting off PH specific therapy with us” –* Physician, Site A

## Discussion

Across three expert PH centers in the United States, we identified notable gaps in the access and timeliness of PH care across the trajectory of a patient’s journey from symptom onset through longitudinal care. Importantly, our findings both address the call from the PH community to incorporate patient perspectives in efforts to improve PH care [26] and reveal specific opportunities to mitigate delays and improve the quality of care for patients living with this devastating disease.

In line with prior studies [18–20], patients described significant delays in receiving a PH diagnosis and reaching expert PH care after the development of symptoms, with tortuous journeys through the healthcare system characterized by dismissal of their symptoms by providers and ill-defined channels to expert care. As highlighted by our participants, the drivers of these diagnostic and referral delays are likely multifactorial, including limited PH-specific knowledge among non-experts and disconnected referral networks.

Prior work has shown that even among practicing cardiologists and pulmonologists, PH is often not considered in patients presenting with symptoms consistent with the disease [27]; these knowledge gaps are likely even greater among general practitioners. Physician postgraduate training programs, including cardiology and pulmonary fellowships, often offer inadequate PH education [28]. As proposed by our participants, active outreach from PH experts to non-expert providers through telementoring models like Project ECHO (Extension for Community Healthcare Outcomes) could help address these training gaps and improve non-expert knowledge of PH [29]. Likewise, creating and disseminating protocols for the structured evaluation of dyspnea as has been previously proposed [30] could promote timely performance of appropriate diagnostic tests and referrals to expert care. Finally, improving the visibility of PH experts and care centers could strengthen referral channels from community providers. Pulmonary hypertension associations have begun this work in the United States and Europe by broadcasting centers capable of providing comprehensive PH care [31, 32]. Likewise, the European Reference Networks for rare diseases includes a PH network that provides and monitors competency requirements for PH centers and disseminates included centers to patient organizations and the public [33]. However, additional outreach activities from PH experts may be needed to foster relationships with community providers and encourage early diagnostic testing and referrals. Furthermore, care networks for PH in resource-limited countries are severely underdeveloped and likely contribute to delayed and poor-quality PH care [34].

We found that geographic barriers to accessing PH care exist at multiple timepoints in the care continuum, from reaching PH care to remaining in care. While the number of accredited PH centers in the US has more than doubled over the past decade [31], the vast majority are located in urban areas [35], and 14 states in the US have no PH centers [36]. Caring for patients with PH in these care deserts remains a significant challenge, which may contribute to higher mortality among patients with PH living in rural areas [35, 37]. Addressing these geographic barriers to access will likely require a multifaceted approach. Expanding the use of provider-to-patient virtual visits within PH centers could facilitate access to PH expertise while allowing patients to remain local [38]. Likewise, establishing satellite sites associated with the PH center could extend PH expertise to surrounding communities and improve access [39, 40]. Finally, building collaborative relationships between PH experts and community providers with clearly defined roles and responsibilities could allow some PH care to be delivered locally and minimize patient burden of traveling to PH centers [31]. This suite of solutions would not only address geographic barriers to care, but could also address economic disparities in PH care that have previously been identified [41, 42]. Importantly, as noted by our participants, PH expert capacity is already limited. Given the rising prevalence of PH [43], shortages of PH experts are likely to worsen. Solutions to improve PH access will need to be coupled with expansion of PH expert availability, which may include expanding PH training programs [28, 44], increasing reimbursement for PH care, and providing PH clinics with more resources and support.

We found that patients experienced significant delays in starting PH therapy due to the high cost of medications and inadequate insurance coverage, with persistent challenges in accessing PH medications even after initiating treatment. Even among patients with health insurance, high deductibles and copayments for PH medications can lead to substantial out-of-pocket costs exceeding $1000 USD per month in the United States [45]. While financial support programs are available through non-profit organizations, many of these programs have long wait-lists or narrow eligibility criteria [46], and applying for these programs can be burdensome for patients as highlighted by our participants. Importantly, we found that these financial barriers often resulted in interruptions in PH therapy, which can have severe consequences such as decline in functional status or right heart failure [47, 48]. Expanding healthcare coverage for patients with PH and improving coverage for PH therapies will be vital to ensure timely access to PH therapies [49]. As has been previously demonstrated, this expansion of coverage could be achieved with minimal overall change in costs to payors given the anticipated reduction in hospitalizations and other healthcare costs [50, 51]. Indeed, minimal or no out-of-pocket costs to patients for PH medications have been achieved in other countries, including resource-limited countries, through national insurance coverage or designation of PH therapies as orphan drugs [52].

While our study focused on pulmonary hypertension, the barriers to timely access to care we identified are likely not unique to PH. Furthermore, the proposed solutions from this work, including expanding use of technology, establishing relationships between expert centers and community providers, and building telementoring models to improve non-expert knowledge can be applied to other complex pulmonary diseases such as interstitial lung disease or sarcoidosis to address access barriers in other contexts [53–55].

Our study has limitations. First, our sample of three US expert PH centers may not be representative of PH care in all expert centers in the US nor of PH care outside the US. For example, two of the three sites predominantly care for underserved patient populations that may not be reflective of all expert centers. Capturing perspectives on PH care access among patients and providers outside the expert center settings and in other countries may shed additional light into these issues. Second, our sample included only 3 patients with CTEPH, a patient group that may experience unique challenges in receiving timely PH care. Third, PH care was managed within the pulmonary departments of the centers included in our study, with only 1 cardiologist involved in PH care across the sites. Including cardiologists in future work may add unique perspectives. Finally, while we achieved the recommended sample size for qualitative research [24], our inability to recruit patients from one site may have affected our results.

## Conclusions

Patients living with pulmonary hypertension experience significant barriers in receiving timely PH care across the entirety of their health journey. Multifaceted interventions on the provider, healthcare system, and health policy levels are needed to improve access to PH care along the PH care continuum and improve outcomes for patients with PH.

## Supplementary Information

Supplementary Material 1.

## Data Availability

Individual interviews are not available publicly to preserve the privacy and anonymity of the participants. However, extracted quotes (coded) from interviews are available in this manuscript.

## Abbreviations

CTEPH: Chronic thromboembolic pulmonary hypertension
PAH: Pulmonary arterial hypertension
PH: Pulmonary hypertension

## References

[1] Humbert M, Kovacs G, Hoeper MM, Badagliacca R, Berger RMF, Brida M, et al. 2022 ESC/ERS guidelines for the diagnosis and treatment of pulmonary hypertension: developed by the task force for the diagnosis and treatment of pulmonary hypertension of the European society of cardiology (ESC) and the European respiratory society (ERS). Endorsed by the international society for heart and lung transplantation (ISHLT) and the European reference network on rare respiratory diseases (ERN-LUNG). Eur Heart J. 2022;43:3618–731.36017548

[2] Kubota K, Miyanaga S, Akao M, Mitsuyoshi K, Iwatani N, Higo K, et al. Association of delayed diagnosis of pulmonary arterial hypertension with its prognosis. J Cardiol. 2023. 10.1016/j.jjcc.2023.08.004. 37579874 10.1016/j.jjcc.2023.08.004

[3] Vizza CD, Badagliacca R, Messick CR, Rao Y, Nelsen AC, Benza RL. The impact of delayed treatment on 6-minute walk distance test in patients with pulmonary arterial hypertension: A meta-analysis. Int J Cardiol. 2018;254:299–301. 29254882 10.1016/j.ijcard.2017.12.016

[4] Frost A, Badesch D, Gibbs JSR, Gopalan D, Khanna D, Manes A, et al. Diagnosis of pulmonary hypertension. Eur Respir J. 2019;53:1801904. 30545972 10.1183/13993003.01904-2018 PMC6351333

[5] McLaughlin VV, Shah SJ, Souza R, Humbert M. Management of pulmonary arterial hypertension. J Am Coll Cardiol. 2015;65:1976–97. 25953750 10.1016/j.jacc.2015.03.540

[6] Chakinala MM, Farber HW. Pulmonary arterial hypertension and specialty care centers: we had a feeling; now we have data. Chest. 2020;158:28–30. 32654707 10.1016/j.chest.2020.03.079

[7] Pi H, Kosanovich CM, Handen A, Tao M, Visina J, Vanspeybroeck G, et al. Outcomes of pulmonary arterial hypertension are improved in a specialty care center. Chest. 2020;158:330–40. 32109446 10.1016/j.chest.2020.01.046 PMC7339236

[8] Wetherill J. Pulmonary Hypertension Awareness Month. Pulmonary Hypertension Association. https://phassociation.org/awarenessmonth/. Accessed 27 Mar 2024.

[9] Didden E-M, Lee E, Wyckmans J, Quinn D, Perchenet L. Time to diagnosis of pulmonary hypertension and diagnostic burden: A retrospective analysis of nationwide US healthcare data. Pulm Circ. 2023;13:e12188. 36694845 10.1002/pul2.12188 PMC9843478

[10] Rich S, Dantzker DR, Ayres SM, Bergofsky EH, Brundage BH, Detre KM, et al. Primary pulmonary hypertension. A National prospective study. Ann Intern Med. 1987;107:216–23. 3605900 10.7326/0003-4819-107-2-216

[11] Kopeć G, Forfia P, Abe K, Beaudet A, Gressin V, Jevnikar M, et al. Recognition, diagnosis, and operability assessment of chronic thromboembolic pulmonary hypertension (CTEPH): A global cross-sectional scientific survey (CLARITY). Pulm Circ. 2024;14:e12330. 38384932 10.1002/pul2.12330 PMC10880430

[12] Deaño RC, Glassner-Kolmin C, Rubenfire M, Frost A, Visovatti S, McLaughlin VV, et al. Referral of patients with pulmonary hypertension diagnoses to tertiary pulmonary hypertension centers: the multicenter repherral study. JAMA Intern Med. 2013;173:887–93. 23568223 10.1001/jamainternmed.2013.319

[13] Saunders H, Helgeson SA, Abdelrahim A, Rottman-Pietrzak K, Reams V, Zeiger TK, et al. Comparing diagnosis and treatment of pulmonary hypertension patients at a pulmonary hypertension center versus community centers. Diseases. 2022;10:5. 35076491 10.3390/diseases10010005 PMC8788556

[14] Gillmeyer KR, Rinne ST, Qian SX, Maron BA, Johnson SW, Klings ES, et al. Socioeconomically disadvantaged veterans experience treatment delays for pulmonary arterial hypertension. Pulm Circ. 2022;12:e12171. 36568691 10.1002/pul2.12171 PMC9768567

[15] Strange G, Gabbay E, Kermeen F, Williams T, Carrington M, Stewart S, et al. Time from symptoms to definitive diagnosis of idiopathic pulmonary arterial hypertension: the delay study. Pulm Circ. 2013;3:89–94. 23662179 10.4103/2045-8932.109919 PMC3641745

[16] Khou V, Anderson JJ, Strange G, Corrigan C, Collins N, Celermajer DS, et al. Diagnostic delay in pulmonary arterial hypertension: insights from the Australian and new Zealand pulmonary hypertension registry. Respirology. 2020;25:863–71. 31997504 10.1111/resp.13768

[17] Ginoux M, Turquier S, Chebib N, Glerant J-C, Traclet J, Philit F, et al. Impact of comorbidities and delay in diagnosis in elderly patients with pulmonary hypertension. ERJ Open Res. 2018;4:00100–2018. 30510957 10.1183/23120541.00100-2018 PMC6258090

[18] Ivarsson B, Johansson A, Kjellström B. The odyssey from symptom to diagnosis of pulmonary hypertension from the patients and spouses perspective. J Prim Care Community Health. 2021;12:21501327211029241. 34219509 10.1177/21501327211029241 PMC8255571

[19] Armstrong I, Rochnia N, Harries C, Bundock S, Yorke J. The trajectory to diagnosis with pulmonary arterial hypertension: a qualitative study. BMJ Open. 2012;2:e000806. 22514243 10.1136/bmjopen-2011-000806 PMC3332243

[20] Alami S, Cottin V, Mouthon L, Desjeux D, Quessette E, Poiraudeau S, et al. Patients’, relatives’, and practitioners’ views of pulmonary arterial hypertension: A qualitative study. La Presse Médicale. 2016;45:e11–27. 26775203 10.1016/j.lpm.2015.06.017

[21] HIV Care Continuum. HIV.gov. https://www.hiv.gov/federal-response/policies-issues/hiv-aids-care-continuum. Accessed 27 Mar 2024.

[22] Palinkas LA, Horwitz SM, Green CA, Wisdom JP, Duan N, Hoagwood K. Purposeful sampling for qualitative data collection and analysis in mixed method implementation research. Adm Policy Ment Health. 2015;42:533–44. 24193818 10.1007/s10488-013-0528-y PMC4012002

[23] Mendez A. Enlisting the gatekeeper: Chain-Referral and elite access in foreign policy analysis. Thousand Oaks: SAGE Publications Ltd; 2020.

[24] Hennink M, Kaiser BN. Sample sizes for saturation in qualitative research: A systematic review of empirical tests. Soc Sci Med. 2022;292:114523. 34785096 10.1016/j.socscimed.2021.114523

[25] Three Approaches to Qualitative Content Analysis - Hsiu-Fang, Hsieh SE, Shannon. 2005. https://journals.sagepub.com/doi/10.1177/1049732305276687. Accessed 5 Oct 2023. 10.1177/1049732305276687 16204405

[26] McGoon MD, Ferrari P, Armstrong I, Denis M, Howard LS, Lowe G, et al. The importance of patient perspectives in pulmonary hypertension. Eur Respir J. 2019;53:1801919. 30545977 10.1183/13993003.01919-2018 PMC6351339

[27] de Belen E, McConnell JW, Elwing JM, Paculdo D, Cabaluna I, Linder J, et al. Gaps in the care of pulmonary hypertension: A Cross-Sectional patient simulation study among practicing cardiologists and pulmonologists. J Am Heart Assoc. 2023;12:e026413. 36628980 10.1161/JAHA.122.026413 PMC9939058

[28] Elliott CG, Barst RJ, Seeger W, Porres-Aguilar M, Brown LM, Zamanian RT, et al. Worldwide physician education and training in pulmonary hypertension: pulmonary vascular disease: the global perspective. Chest. 2010;137:S85–94. 10.1378/chest.09-2816 20522584

[29] Osei-Twum J-A, Wiles B, Killackey T, Mahood Q, Lalloo C, Stinson JN. Impact of project ECHO on patient and community health outcomes: A scoping review. Acad Med. 2022;97:1393–402. 35612913 10.1097/ACM.0000000000004749

[30] Ahmed S, Ahmed A, Rådegran G. Structured evaluation of unclear dyspnea-An attempt to shorten the diagnostic delay in pulmonary arterial hypertension and chronic thromboembolic pulmonary hypertension. Pulm Circ. 2024;14:e12340. 38317859 10.1002/pul2.12340 PMC10839287

[31] Chakinala MM, Duncan M, Wirth J. Managing the patient with pulmonary hypertension: specialty care centers, coordinated care, and patient support. Cardiol Clin. 2016;34:489–500. 27443143 10.1016/j.ccl.2016.04.008

[32] Call to Action on the Unmet Needs of Patients with Pulmonary Hypertension.– 2012. PHA Europe. 2024. https://www.phaeurope.org/get-involved/advocacy-policy-work/call-to-action-on-the-unmet-needs-of-patients-with-pulmonary-hypertension-2012/. Accessed 24 Oct 2024.

[33] ERN-LUNG - Pulmonary hypertension (PH). ERS Respiratory Channel. https://channel.ersnet.org/\\channel.ersnet.org/fo-channel-home.php?id=160&contId. Accessed 24 Oct 2024.

[34] Lopes AA, Bandeira AP, Flores PC, Tavares Santana MV. Pulmonary hypertension in Latin america: pulmonary vascular disease: the global perspective. Chest. 2010;137:S78–84. 10.1378/chest.09-2960 20522583

[35] Issa R, Minhas AMK, Issa R, Ariss RW, Nazir S, Satti DI, et al. Urban-Rural disparities in pulmonary Hypertension-Related mortality between 2004 and 2019: A call to improve access to specialty care centers for rural residents in the united States. Curr Probl Cardiol. 2023;48:101623. 36731687 10.1016/j.cpcardiol.2023.101623

[36] PHA A. PH Care Centers - Accredited Centers. Pulmonary Hypertension Association. https://phassociation.org/phcarecenters/accredited-centers/. Accessed 3 Apr 2024.

[37] Macías CG, Wharam JF, Maron BA, Ong M-S. Urban–Rural disparities in pulmonary hypertension mortality. Annals ATS. 2020;17:1168–71. 10.1513/AnnalsATS.202003-234RL 32584607

[38] Butzner M, Cuffee Y. Telehealth interventions and outcomes across rural communities in the united states: narrative review. J Med Internet Res. 2021;23:e29575. 34435965 10.2196/29575 PMC8430850

[39] Wood BR, Bell C, Carr J, Aleshire R, Behrens CB, Dunaway SB, et al. Washington state satellite HIV clinic program: a model for delivering highly effective decentralized care in under-resourced communities. AIDS Care. 2018;30:1120–7. 29852744 10.1080/09540121.2018.1481194 PMC6334292

[40] Katz DA, Mohan S, Bacon M, McGovern E, Wallen WJ, Preston GM, et al. Regionalization or access to care?? A joint pediatric heart care? program that achieves both: one program-Two sites. World J Pediatr Congenit Heart Surg. 2023;14:155–60. 36866598 10.1177/21501351221149420

[41] Talwar A, Sahni S, Talwar A, Kohn N, Klinger JR. Socioeconomic status affects pulmonary hypertension disease severity at time of first evaluation. Pulmonary Circulation. 2016;6:191–5. 27252845 10.1086/686489 PMC4869923

[42] Wu W-H, Yang L, Peng F-H, Yao J, Zou L-L, Liu D, et al. Lower socioeconomic status is associated with worse outcomes in pulmonary arterial hypertension. Am J Respir Crit Care Med. 2013;187:303–10. 23220911 10.1164/rccm.201207-1290OC PMC3603556

[43] Wijeratne DT, Lajkosz K, Brogly SB, Lougheed MD, Jiang L, Housin A, et al. Increasing incidence and prevalence of world health organization groups 1 to 4 pulmonary hypertension: A Population-Based cohort study in ontario, Canada. Circ Cardiovasc Qual Outcomes. 2018;11:e003973. 29444925 10.1161/CIRCOUTCOMES.117.003973 PMC5819352

[44] Kudelko K, Zamanian RT, De Jesus Perez VA. Pulmonary Vascular Fellowship Training to Promote Excellence in PH Clinical Care: The Stanford Perspective. 2020. https://www.acc.org/latest-in-cardiology/articles/2020/09/21/19/13/pulmonary-vascular-fellowship-training-to-promote-excellence-in-ph-clinical-care. Accessed 3 Apr 2024.

[45] Helgeson SA, Menon D, Helmi H, Vadlamudi C, Moss JE, Zeiger TK, et al. Psychosocial and financial burden of therapy in USA patients with pulmonary arterial hypertension. Diseases. 2020;8:22. 32545763 10.3390/diseases8020022 PMC7349780

[46] Wetherill J. Apr. Financial Assistance. Pulmonary Hypertension Association. https://phassociation.org/patients/insurance-and-treatment-access/financialassist/. Accessed 3 Apr 2024.

[47] Narechania S, Torbic H, Tonelli AR. Treatment discontinuation or interruption in pulmonary arterial hypertension. J Cardiovasc Pharmacol Ther. 2020;25:131–41. 31594400 10.1177/1074248419877409

[48] Murali S, Khanal S, Banerjee S, Christie O, Ramakrishna K. Pause at your own peril: A case series on rebound pulmonary hypertension. Cureus. 2022;14:e25552. 35783883 10.7759/cureus.25552 PMC9249055

[49] Highland KB, Hughes KE, Williams KJ, Kyei-Baffour B, Ferguson S. Ensuring appropriate access to pulmonary arterial hypertension therapy. Am J Manag Care. 2019;25(7 Suppl):S119–27. 31318517

[50] Burger CD, Ghandour M, Padmanabhan Menon D, Helmi H, Benza RL. Early intervention in the management of pulmonary arterial hypertension: clinical and economic outcomes. Clinicoecon Outcomes Res. 2017;9:731–9. 29200882 10.2147/CEOR.S119117 PMC5703162

[51] Burger CD, Ozbay AB, Lazarus HM, Riehle E, Montejano LB, Lenhart G, et al. Treatment patterns and associated health care costs before and after treatment initiation among pulmonary arterial hypertension patients in the united States. J Manag Care Spec Pharm. 2018;24:834–42. 29436260 10.18553/jmcp.2018.17391 PMC10398102

[52] Orozco-Levi M, Cáneva J, Fernandes C, Restrepo-Jaramillo R, Zayas N, Conde R, et al. Differences in health policies for drug availability in pulmonary arterial hypertension and chronic thromboembolic pulmonary hypertension across Latin America. Pulmonary Circulation. 2022;12:e12012. 35506085 10.1002/pul2.12012 PMC9053007

[53] Pritchard D, Adegunsoye A, Lafond E, Pugashetti JV, DiGeronimo R, Boctor N, et al. Diagnostic test interpretation and referral delay in patients with interstitial lung disease. Respir Res. 2019;20:253. 31718645 10.1186/s12931-019-1228-2 PMC6852922

[54] Johannson KA, Lethebe BC, Assayag D, Fisher JH, Kolb M, Morisset J, et al. Travel distance to subspecialty clinic and outcomes in patients with fibrotic interstitial lung disease. Ann Am Thorac Soc. 2022;19:20–7. 34033739 10.1513/AnnalsATS.202102-216OC

[55] Gerke AK, Judson MA, Cozier YC, Culver DA, Koth LL. Disease burden and variability in sarcoidosis. Ann Am Thorac Soc. 2017;14(Suppl 6):S421–8. 29087725 10.1513/AnnalsATS.201707-564OT PMC5802572

