# Supplementary material for: Gaps in access to pulmonary hypertension care and opportunities for improvement: a multi-site qualitative study
Source: BMC Pulm Med. 2025 Jul 28;25:355. doi: 10.1186/s12890-025-03817-4 (PMC12302599; doi:10.1186/s12890-025-03817-4)
Supplement: Supplementary file 1 — Supplementary Material 1. [file 12890_2025_3817_MOESM1_ESM.docx]

**ONLINE SUPPLEMENT**

**Title:** Gaps in access to pulmonary hypertension care and opportunities for improvement: a multi-site qualitative study

**Authors:** Kari R. Gillmeyer, MD, MSc^1,2^; Sara Shusterman, MD^1,2^; Seppo T. Rinne, MD, PhD^1-3^; A. Rani Elwy, PhD^1,4^; Renda Soylemez Wiener, MD, MPH^1,2^

**Interview Guide for Patients**

**Symptom onset / reaching PH care**

Tell me about your experience with receiving a diagnosis of pulmonary hypertension.

*Probes:*

- *When were you first diagnosed with pulmonary hypertension?*
- *How many providers did you see after your symptoms started before you received a diagnosis of pulmonary hypertension?*
- *What kinds of testing did you have before you were diagnosed?*
- *What types of different doctors have you seen for your pulmonary hypertension?*

Tell me about your experience with being referred to pulmonary hypertension specialists at [PH center]?

*Probes:*

- *Were the specialists in the same clinic / hospital as your primary care physician or did you need to see a specialist at a different hospital?*
- *Were there any challenges in getting the referral?*
- *How long did it take to get the referral?*
- *How did you find the PH specialist / PH center?*

**Diagnosis / treatment within the PH center**

- How long have you been receiving care at [PH center]?
- What has your medical care been like at [PH center]?
- Are you on any treatment for pulmonary hypertension? What was your experience with being started on treatment?

*Probes:*

- *Did you experience in challenges or delays in starting treatment?*
- *Did you work with any financial support programs? What was that experience like?*

**Long-term follow-up**

- How many doctors/providers do you currently see for your pulmonary hypertension?
- How often do you see doctors/providers for pulmonary hypertension? In what clinics/hospitals?
- Do you ever have difficulty getting in to see your doctors/providers for regular follow-up visits? In the case of an urgent or emergent issue?
- If you can’t get in to see your PH doctors/providers in a timely fashion, where do you seek medical care?
- Do you have access to the medications you need to treat your pulmonary hypertension?

**Interview Guide for Providers**

**Reaching PH care**

Can you tell me the ways that patients reach PH care at your center?

*Probes:*

- *For example, are patients referred from other providers (e.g., primary care, other specialties) at [your institution]? Are patients referred in from other institutions or health centers? What is the breakdown of those referrals?*
- *Are there any challenges or barriers in the referral process?*

**Diagnosis / treatment within the PH center**

What structures or resources are available to facilitate PH care at your center?

*Probes:*

- *For example, do you have advanced practice providers, clinical nurse specialists, patient navigators? What are their specific roles?*
- *Do you have dedicated pharmacy support for PH care? What are the specific roles of pharmacists?*

What barriers (if any) exist for patients to access pulmonary care? Are there structures or practices that impede access to PH care?

What existing systems (if any) exist to facilitate rapid access to PH care? How do scheduling practices facilitate more rapid access to care?

When it comes to PH care, what does your center do particularly well?

What are some of the challenges of providing PH care at your center?

**Long-term follow-up**

Can you describe how patients with PH are followed longitudinally?

*Probes:*

- *For example, how are follow-up visits or pending tests tracked? How are individuals “lost-to-follow-up” identified?*
- *Are patients ever “returned” to their referring provider for long-term followed-up? How does that transition happen?*

What barriers (if any) exist in the longitudinal care of patients with PH?
